# Critical care ultrasound: development, evolution, current and evolving clinical concepts in critical care medicine

**DOI:** 10.3389/fmed.2025.1622604

**Published:** 2025-08-06

**Authors:** Jing Su, Xin Tie, Ying Wei, Ran Zhou, Tongjuan Zou, Yao Qin, Xueying Zeng, Yi Li, Wanhong Yin

**Affiliations:** Department of Critical Care Medicine, West China Hospital, Sichuan University, Chengdu, China

**Keywords:** ultrasound, critical care, development, evolution, challenges

## Abstract

Critical care ultrasound (CCUS) has emerged as a vital tool in modern critical care medicine (CCM), revolutionizing the assessment and management of critically ill patients. CCUS provides real-time insights into patient conditions and enables physicians to analyze the underlying mechanisms and pathophysiology of critical illnesses based on established theories and clinical needs, ultimately visualizing the phenotypes of patients and guiding clinical practice. The innovations of wearable ultrasound and the incorporation of artificial intelligence are further reshaping and broadening its application. This review underscores the importance of CCUS as an integral component of CCM, highlighting its development, current applications, and future directions. In addition, questions are raised regarding the standard training and guidelines of CCUS needing to be addressed in the near future.

## 1 Introduction

In recent years, critical care medicine (CCM) has undergone rapid advancements. These developments are evident not only in the updates to diagnostic and therapeutic concepts and techniques but also in the increasing demand from clinicians for dynamic assessment and monitoring of patient’s conditions. In this context, critical care ultrasound (CCUS) has emerged. CCUS is a problem-oriented and dynamic evaluation process that employs ultrasound technology, guided by the principles of CCM, to address specific issues in critically ill patients through a multi-targeted and integrated approach (1). It is essential for determining treatment direction, particularly in guiding hemodynamic management and enabling precise adjustments. CCUS represents a significant integration of CCM with ultrasound technology. This article explores the development and evolution of CCUS within the field of CCM.

## 2 Methods

### 2.1 Eligibility criteria

The inclusion criteria were as follows: (1) peer-reviewed original articles, systematic reviews, meta-analyses, evidence-based guidelines, and clinical protocols; (2) publications dated prior to November 2024; (3) publications focused on the history of the development and evolution of CCUS, clinical applications in CCM, advanced wearable ultrasound technologies, artificial intelligence integration in ultrasound, and standardized training methodologies. The exclusion criteria were as follows: (1) case reports, editorials, or non-English publications without validated translations; (2) studies not directly applicable to emergency or intensive care clinical contexts.

### 2.2 Information sources and search strategy

This review was conducted through a systematic search of PubMed, Web of Science, Embase, and China National Knowledge Infrastructure (CNKI) databases. Our search strategy incorporated both Medical Subject Headings (MeSH) and free-text keywords, structured into four conceptual domains: (1) Critical care concepts: “intensive care” OR “critical care” OR “intensive care unit” OR “ICU” OR “emergency medicine”; (2) Ultrasound terminology: “point-of-care ultrasound” OR “POCUS” OR “critical care ultrasound” OR “CCUS” OR “bedside ultrasound” OR “echocardiography”; (3) Technology innovations: “artificial intelligence” OR “deep learning” OR “machine learning” OR “wearable devices” OR “wearable ultrasound”; (4) Clinical applications: “diagnosis” OR “monitor” OR “phenotype” OR “procedural guidance”.

### 2.3 Selection process

Two independent reviewers screened all identified articles for relevance. Discrepancies were resolved through consensus discussion or, when required, by consultation with a third reviewer. For eligible studies, both reviewers independently extracted data using a standardized form that included: (1) study characteristics: authors, publication year, study design, and sample size; (2) CCUS applications: diagnostic uses, monitoring approaches, and procedural guidance; (3) technological specifications: AI algorithms employed, and device types utilized; (4) clinical outcomes: diagnostic accuracy, and measured clinical impact; and (5) study limitations with potential biases.

## 3 From traditional ultrasound to critical care ultrasound

### 3.1 Traditional ultrasound: laying the foundation

In 1942, Austrian neurologist Karl Theodore Dussik reported the first case of using ultrasound technology to diagnose a brain tumor, marking the beginning of the medical ultrasound era (2). In the 1950s, a research team led by Ian Donald developed the first medical diagnostic ultrasound device (3). Through the dedicated efforts of professionals across diverse disciplines, including medicine, engineering, and electronics, ultrasound technology has achieved milestone breakthroughs. Its non-invasive nature, real-time imaging capabilities, and cost-effectiveness have made it an indispensable tool in clinical practice (4). In routine diagnostic workflows, ultrasound examinations are generally conducted in radiology departments by radiologists or cardiologists who have undergone rigorous training. However, this process involves several sequential steps, including clinical reception, request issuance, image acquisition, report writing, and review. Consequently, the information provided by traditional ultrasound examinations can be somewhat delayed, which poses challenges in addressing the urgent need for immediate evaluation in critical scenarios.

### 3.2 Point of care ultrasound: revolutionizing bedside care

Between the 1980s and 1990s, real-time imaging and Doppler ultrasound technology underwent significant advancements, and more portable and accurate ultrasound equipment was developed and popularized. These innovations facilitated the transition of ultrasound technology from a specialized domain to routine use at the bedside (4–7). Point-of-care ultrasound (POCUS), also known as targeted, clinical, or goal-directed ultrasound examination, has been extensively utilized in emergency medicine and CCM since the 1990s (8). In 1990, the American College of Emergency Physicians (ACEP) published a POCUS statement delineating five key areas of its practice: resuscitation, diagnosis, symptom or sign-based evaluation, procedural guidance, therapeutic and monitoring (9). As clinicians perform ultrasound examinations directly, the focus of assessments and image interpretations closely aligns with clinical needs. POCUS enables healthcare teams to acquire real-time bedside imaging for rapid clinical assessment in high-risk environments and aids clinicians in safely performing invasive procedures. This capability allows for timely adjustments to examination and treatment strategies in response to the various stages of disease progression, thereby revolutionizing bedside care.

### 3.3 Critical care ultrasound: advancing structured assessment

Between 1995 and 2009, Daniel Lichtenstein published a series of pioneering studies on applying lung ultrasound (LUS) techniques in diagnosing and treating critically ill patients (10–15). During this transformative period, the practice of ultrasound examination evolved considerably, shifting from a narrow focus on evaluating specific organs or anatomical sites to a more integrative approach that encompasses multiple regions of interest. This evolution led to the development of comprehensive and systematic ultrasound protocols, enhancing the overall diagnostic capabilities in critical care settings.

**Figure 1:**
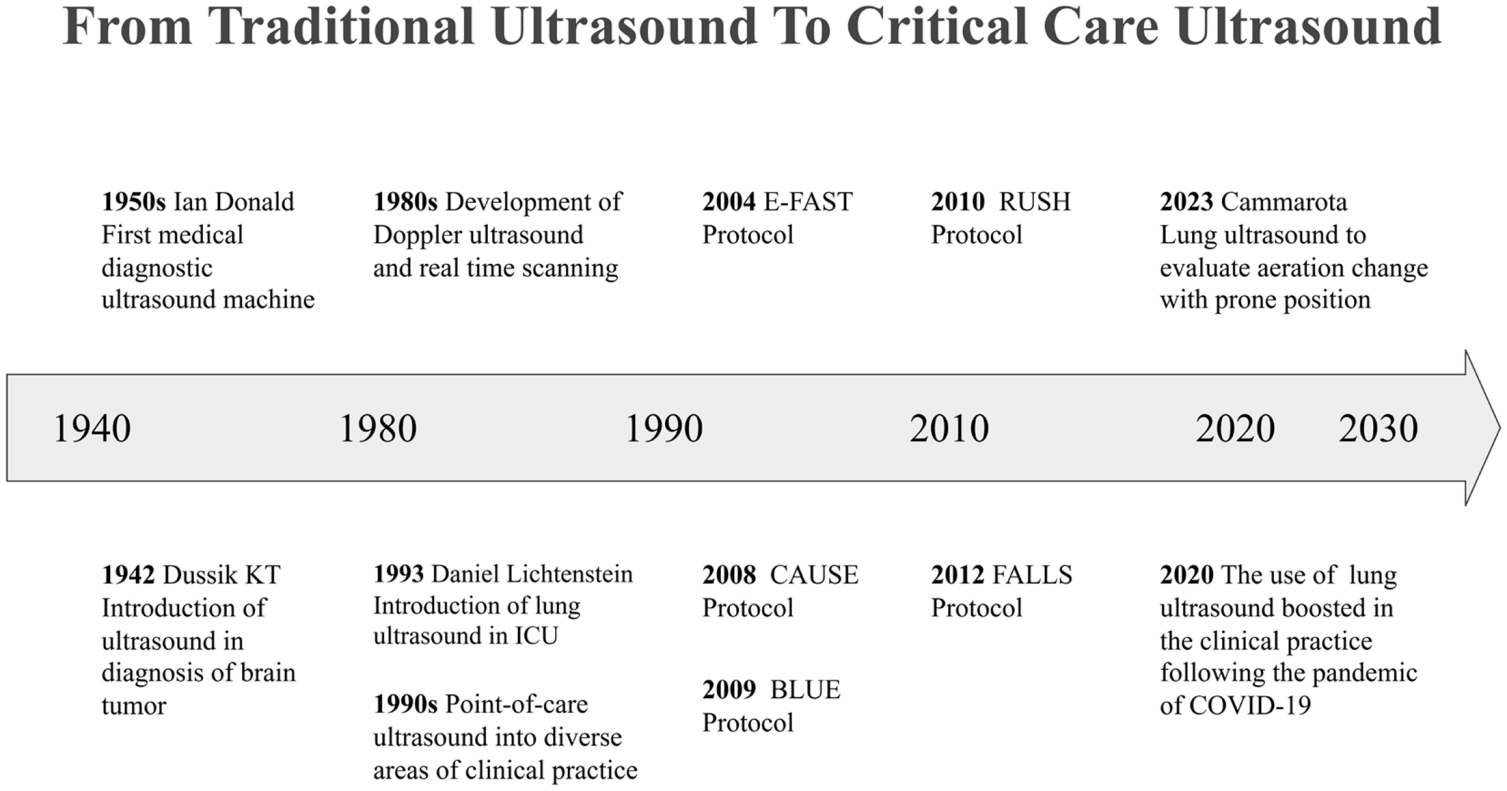
Critical care ultrasound evolution.

One of the foundational advancements in this field was introduced in the 1990s by Rozycki et al., who developed the focused assessment with sonography for trauma (FAST) protocol. This innovative approach was initially designed for the rapid evaluation of trauma patients which was later expanded to include the assessment of pneumothorax, resulting in the Extended FAST (E-FAST) protocol (16–18). Subsequently, ultrasound examination protocols tailored to various clinical scenarios were progressively developed and refined. Notable examples include the bedside lung ultrasound in emergency (BLUE) protocol, which is utilized for the assessment of acute respiratory failure and hypoxemia (10, 15); the rapid ultrasound in shock (RUSH) protocol, aimed at evaluating patients in shock (19); the cardiac arrest ultrasound exam (CAUSE), which assists in the assessment of patients experiencing cardiac arrest (20); and the fluid administration limited by lung sonography (FALLS), designed to guide fluid management in critically ill patients (21). These groundbreaking studies enriched the application of ultrasound examinations in clinical practice and advanced the field of CCUS by promoting structured and modular assessment strategies. The importance of CCUS has become particularly evident in the context of global health crises, such as the COVID-19 pandemic. The implementation of level-3 protection requirements in intensive care units (ICU) further underscored the necessity of CCUS (22, 23) (Figure 1).

In the past few decades, CCUS has undergone a paradigm shift from a tool for diagnosis and procedural assistance to monitoring, moving its focus from a qualitative toward a quantitative assessment of pathologic processes (24). The dynamic nature of critical illness necessitates frequent bedside evaluations, as conditions can deteriorate rapidly. Prompt recognition of these changes is crucial, as timely therapeutic interventions can significantly impact patient outcomes. CCUS has emerged as an invaluable asset in this context, offering high-frequency dynamic monitoring that enables clinicians to closely track patients’ conditions in real-time. By providing critical information such as changes in hemodynamic parameters following interventions, CCUS allows healthcare providers to evaluate the effectiveness of treatments and adjust their strategies accordingly. Furthermore, it aids in analyzing the underlying mechanisms and pathogenesis associated with various critical illnesses. This comprehensive approach not only enhances the understanding of patient conditions but also plays a pivotal role in guiding clinical practice, ensuring that interventions are both timely and appropriate. As a result, CCUS has solidified its position as an essential tool in the critical care setting, contributing significantly to improved patient management and outcomes.

## 4 Visualization of critical illness

The foundation of ultrasound-guided clinical practice in critical care lies in the ability to identify specific phenotypes of pathophysiological disorders and their underlying mechanisms. This understanding is crucial for informing subsequent organ support, protection, and etiological treatment. The process of visualizing critical illness involves three key steps: First, abnormalities in ultrasound images must be identified, including structural, morphological, hemodynamic, and motion-related anomalies. Second, the information represented behind the images should be interpreted and summarized, including pathophysiological changes, host responses, iatrogenic injuries, primary causes, and underlying conditions. Finally, multidimensional sonographic findings should be integrated with clinical data to construct a comprehensive phenotype of the pathophysiological disorders. The “Tripartite List” method proposed by Qin et al. provides a framework for clinicians to systematically organize objective abnormalities along established pathways. This structured approach aids clinicians in identifying the most critical issues within complex clinical contexts and in formulating targeted strategies (25) (Table 1).

**Table 1:** Tripartite list for visualizing critical illness.

Module	Step 1: Examination			Step 2: Interpretation	Step 3: Phenotype
Intravenous volume	Inferior vena cava	Morphology and diameter of the long axis		(1) Venous volume overload (2) Venous volume intermediate state (3) Venous volume deficit	Hemodynamic phenotype: (1) Macrocirculatory hemodynamic phenotype (2) Microcirculatory hemodynamic phenotype (3) Organ hemodynamic phenotype
		Morphology and diameter of the short axis			
		Respiratory variability			
	Hepatic vein	Doppler systolic peak and diastolic peak			
	Portal vein	Pulsatility index			
	Renal vein	Doppler blood flow continuity			
Heart	Chambers	Size (pericardium, atrium, ventricle)		Cardiac pump function: (1) Underlying disease (2) Volume status and responsiveness (3) Right heart function (4) Left heart diastolic function (5) Left heart systolic function Ventriculoarterial coupling: (1) High output with low vascular tone (hyperdynamic shock) (2) Low output with high resistance (hypodynamic shock) (3) Low output with low vascular tone) (1) Normal tissue perfusion (2) Low tissue perfusion (3) Non-perfusion (1) Acute or Chronic (2) Diffuse or limited (3) Unilateral or Bilateral (4) Gravity-dependent or non-gravity-dependent (5) Normal ventilation or hyperventilation or hypoventilation	Pulmonary pathophysiological abnormalities: (1) Interstitial syndrome (2) Focal deaeration (3) Atelectasis of non-gravity-dependent regions (4) Atelectasis of gravity-dependent regions Host-organ unregulated response: (1) Stress disorder (increased respiratory and circulatory drive, takotsubo syndrome) (2) Inflammatory response (vasoplegic syndrome, vascular leak) Primary disease: (1) Focus of infection (2) Trauma (3) Bleeding Secondary changes in chronic disease: (1) Hypertension (2) Chronic obstructive pulmonary disease (3) Chronic kidney disease Iatrogenic wound: (1) Fluid overload (2) Hypostatic pneumonia
		Morphology			
		Ratio of size			
	Walls	Thickness			
		Motion	Hyperkinetic		
			Hypokinetic		
			Incoordination		
			Segmental abnormality		
	Valves	Stenosis			
		Insufficiency			
		Vegetation			
	Flow	Direction	Antegrade flow		
			Regurgitation		
			Shunt		
		Velocity			
		Spectrum analysis			
Peripheral resistance	Snuff-box Resistive Index (SBRI)				
Tissue Perfusion	Renal Doppler Resistive Index (RDRI)	Clearly visible diastolic blood flow			
		Decreased diastolic blood flow			
		Blood flow is visible only during the systolic phase of a cardiac cycle			
Lung	Twelve zones	A-lines			
		B-lines			
		Shred sign			
		Tissue-like pattern			
		Air bronchogram			
		Pleural effusions			
		Lung sliding			
		Seashore sign			
		Lung pulse			
		Lung point			

The ultrasound-guided six-step shock assessment is an example of how hemodynamic principles can be applied to evaluate various aspects of shock. By evaluating the inferior vena cava, right heart function, left ventricular systolic and diastolic function, vascular tone, and tissue perfusion, clinicians can gain a comprehensive understanding of a patient’s volume status, preload and volume responsiveness, cardiac pump function, peripheral vascular resistance, and peripheral perfusion (26). This methodology supports the visualization of hemodynamic phenotypes, enhancing overall shock management in clinical practice. Similarly, the 12-zone LUS protocol allows for the visualization of water-gas ratio characteristics across the lung (23, 27). Wang et al. used machine learning to construct seven pulmonary ultrasound phenotypes covering both gravity-dependent and non-gravity-dependent regions based on the 12-zone imaging and further explored the proportion of each phenotype in severe pneumonia, ARDS, and cardiogenic pulmonary edema (28). However, as a single-center study, its findings were constrained by the quality and diversity of the training data. The identified phenotypes lacked external validation, which may compromise their reproducibility.

## 5 Clinical application of critical care ultrasound in ICU

### 5.1 See first, then intervene

The concept of “see first, then intervene” underscores the importance of visualizing the underlying pathology before initiating treatment. For example, when encountering circulatory failure, it is important to differentiate the type of shock the patient is experiencing, as circulatory failure is categorized into four types of shock: obstructive, cardiogenic, distributive, and hypovolemic, each of which must be treated differently (29). Moreover, patients in the ICU often present with multifactorial conditions, sometimes multiple shocks can overlap, making diagnosis and treatment difficult. CCUS provides real-time, bedside imaging that allows clinicians to directly observe the physiological and pathological changes in critically ill patients. This immediate visual feedback enables more accurate diagnoses and tailored therapeutic strategies.

### 5.2 Rapid stabilization of vital signs

**Figure 2:**
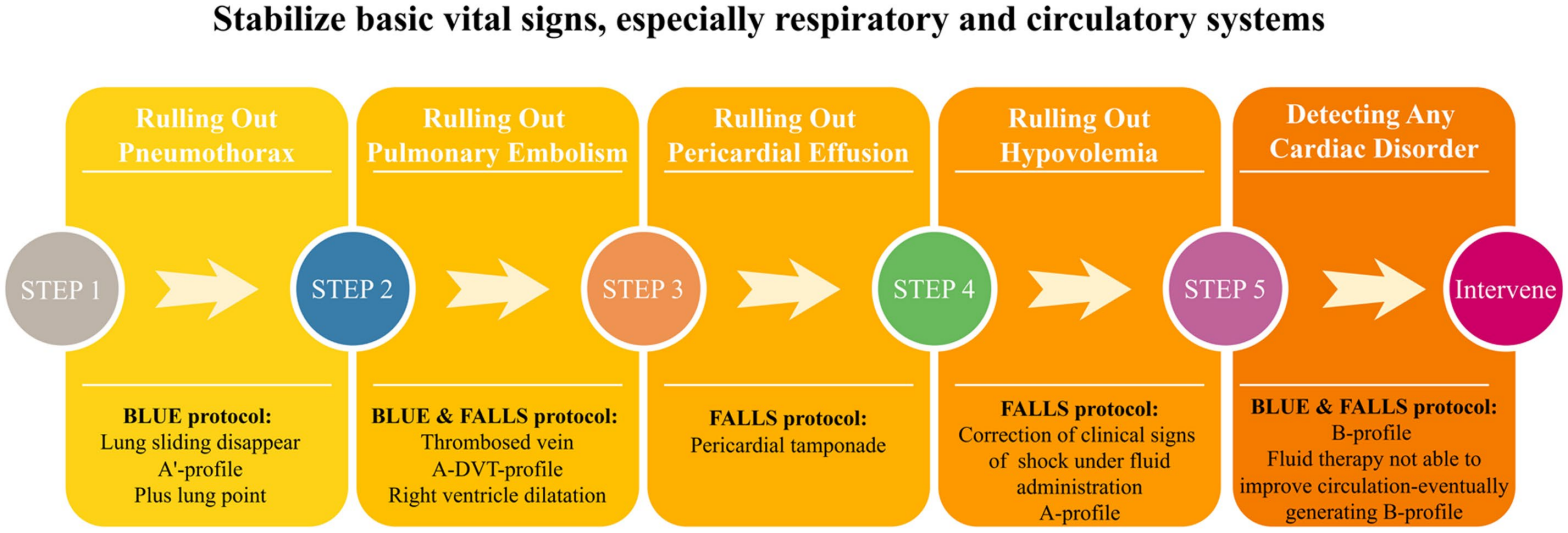
Bedside ultrasound evaluation of acute respiratory and circulatory failure.

The characteristics of critical illnesses necessitate a more rapid response and higher quality management of critically ill patients. Traditional clinical practice often relies on previously obtained imaging results and static laboratory indicators, which may not be enough when a patient’s condition changes. With the CCU, clinicians can obtain dynamic, real-time, and direct insights into anatomical structures and organ function. This enables clinicians to identify life-threatening and reversible conditions and execute timely and safe interventions. For example, combining the BLUE and FALLS protocols effectively addresses the diagnostic and therapeutic needs associated with acute respiratory and circulatory failure (Figure 2). Integrating CCU into routine patient reception and ward rounds reduces redundancy in various clinical data and decision-making time, enhances the quality and efficiency of clinical practice, optimizes the utilization of medical resources, and facilitates rapid stabilization of patients’ vital signs.

### 5.3 Critical care ultrasound in ward rounds

**Figure 3:**
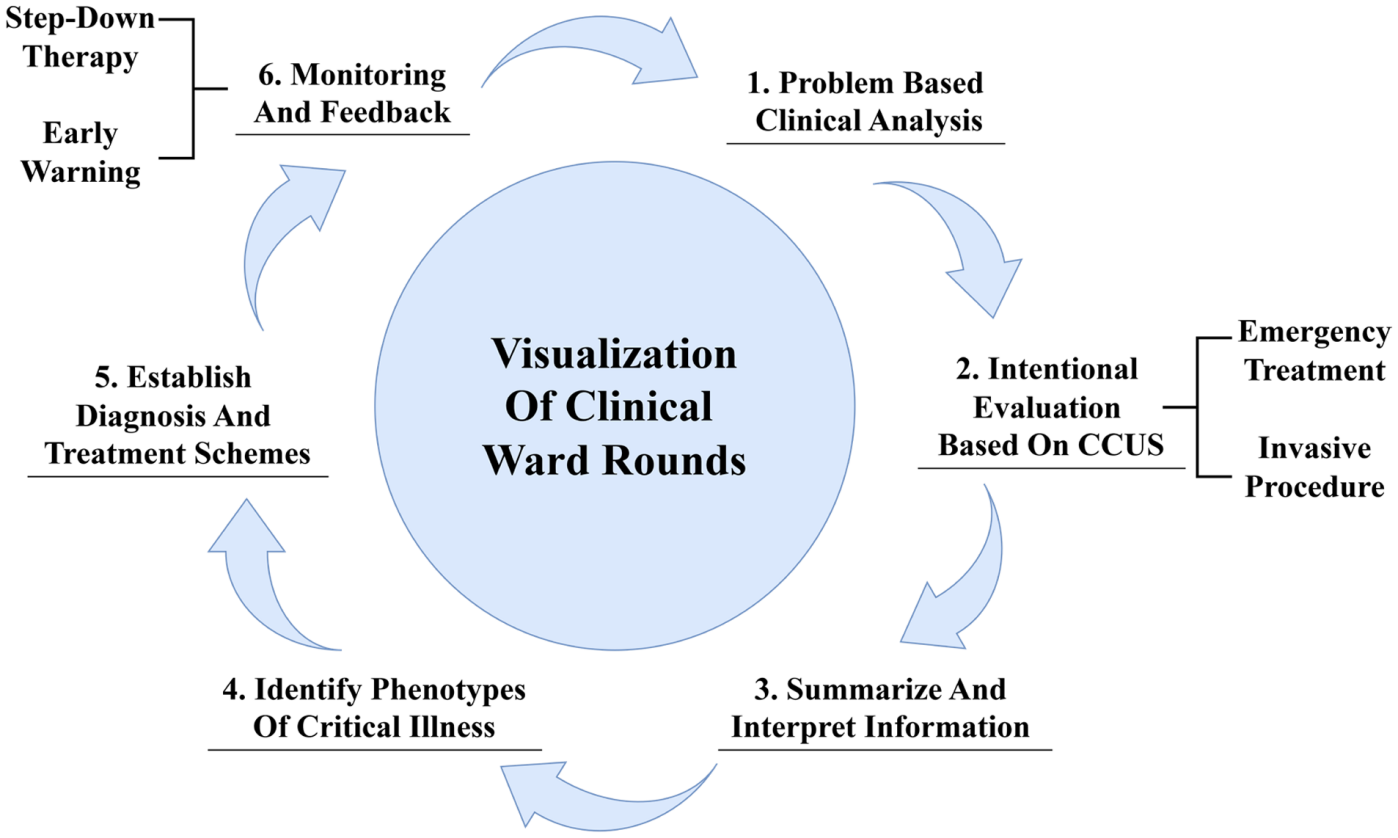
Critical care ultrasound in ward rounds.

The visualization of clinical ward rounds based on CCU aims at the intentional collection of ultrasound information relevant to specific clinical problems, rapid identification of etiology, urgent interventions to stabilize the patient’s vital signs, and observation and re-evaluation of treatment outcomes, thereby enabling personalized and precise care (30). First, clinicians should conduct a systematic review and analysis of existing data to summarize the primary and synergistic clinical problems. Second, they should establish the objectives and develop a protocol for subsequent ultrasound examinations, selectively acquiring key ultrasound information that directly addresses clinical issues. Clinicians are also expected to focus on screening for etiologies and, when necessary, perform invasive procedures under ultrasound guidance. The third step involves a comprehensive interpretation of the core issues and relevant ultrasound images, allowing for a deeper exploration of the pathophysiological changes and their associations with underlying diseases, primary conditions, and iatrogenic injuries. The fourth step involves constructing phenotypes of critical illness using the previously described “Tripartite List” method. In the fifth step, a hierarchical treatment plan and corresponding objectives are developed, including treating underlying etiologies, managing dysregulated host responses, and supporting and protecting affected organs according to the identified phenotypes. Finally, acquiring feedback and making timely adjustments such as stepped-down treatment and rehabilitation training is crucial. Additionally, the integration of CCUS with electronic health records can facilitate data sharing and improve the continuity of care, ensuring a safe transition for the patient through various stages of treatment (Figure 3).

## 6 Ongoing changes in critical care ultrasound

### 6.1 Wearable ultrasound and continuous monitoring

Advancements in microelectronics technology and materials science have led to breakthroughs in the miniaturization of ultrasonic imaging equipment. Wearable ultrasound technology enhances the convenience and flexibility of data collection, facilitating continuous monitoring and tracking of organ function within the intensive care unit. Furthermore, this technology contributes to a deeper understanding of complex pathophysiological processes, thereby advancing personalized and precision medicine.

In 2017, Liu, et al. introduced the concept of “continuous echocardiographic monitoring” in the *Journal Anesthesia & Analgesia* (31). This team developed a wearable color Doppler ultrasound diagnostic instrument that can be utilized in various clinical scenarios, including the continuous monitoring of cardiac function in patients with heart failure, the ultrasound-guided monitoring during minimally invasive cardiac surgeries, the ongoing assessment of patients in shock, and the pre-hospital emergency treatment and transport of critically ill patients. This innovation addresses the limitations of traditional ultrasound probes, which cannot maintain fixed imaging sections for continuous monitoring. Xu, et al. improved the mechanical coupling between the device and human skin through innovations in device design and material fabrication, allowing the evaluation of cardiac function from different views during motion. They also developed a deep learning model that automatically extracts the left ventricular volume from the continuous image recording, yielding waveforms of key cardiac performance indices such as stroke volume, cardiac output, and ejection fraction (32). Apart from cardiac function monitoring, the bioadhesive ultrasound device developed by Zhao, et al. can provide 48 h of continuous imaging of diverse internal organs, including blood vessels, muscle, heart, gastrointestinal tract, diaphragm, and lung, thereby facilitating long-term multi-organ function monitoring (33). Subsequently, this team also developed a novel wearable bioadhesive ultrasound shear wave elastography, which can continuously monitor liver stiffness in a rat model of acute liver failure (34). This achievement is expected to assist organ transplantation monitoring in the ICU.

The development of patch-based ultrasound has made it possible to continuously and autonomously monitor physiological signals from deep tissues. In 2018, Xu, et al. published an ultrasonic device that is conformal to the skin in *Nature Biomedical Engineering*. This device is capable of capturing blood-pressure waveforms at deeply embedded arterial and venous sites and enabling the non-invasive, continuous, and accurate monitoring of cardiovascular events from multiple body locations (35). In 2024, the team reported a fully integrated autonomous wearable ultrasonic-system-on-patch with machine learning designed to track moving tissue targets and assist in data interpretation, which can continuously monitor physiological signals, including central blood pressure, heart rate, and cardiac output, for as long as 12-h (36). The same year, another article detailed a conformal ultrasound patch for hands-free volumetric imaging and continuous monitoring of cerebral blood flow during different interventions. This patch can also identify intracranial B waves during drowsiness (37). Accurate and continuous monitoring of cerebral blood flow is of great value for the diagnosis and treatment of neurocritical care patients, as well as for fundamental research in neurovascular dynamics.

While innovative wearable ultrasound and continuous monitoring technologies show promise, most evidence originates from early-stage feasibility studies. Therefore, there are still some limitations. Most of the research subjects were healthy volunteers or animal models (32–37), and there was a lack of research data on actual patients and clinical outcomes, such as mortality reduction or workflow efficiency (31). While recent technological advances have simplified ultrasound acquisition, operator expertise remains crucial for probe positioning and angle optimization, image interpretation, and quality control. It was not reported in detail whether the operational level and technical differences would affect the measurement results. Moreover, technological disparity risks exacerbating global healthcare inequalities, as resource-limited institutions struggle to adopt these innovations (Table 2).

**Table 2:** Revolutionary applications of wearable ultrasound devices.

Year	Researcher	Contribution
2017	Jin Liu (31)	Tailored holder for continuous echocardiographic monitoring.
2018	Sheng Xu (35)	Monitoring of the central blood pressure waveform via a conformal ultrasonic device.
2022	Xuanhe Zhao (33)	Bioadhesive ultrasound for long-term continuous imaging of diverse organs.
2023	Sheng Xu (32)	A wearable cardiac ultrasound imager.
2024	Sheng Xu (36)	A fully integrated wearable ultrasound system to monitor deep tissues in moving subjects.
2024	Xuanhe Zhao (34)	Wearable bioadhesive ultrasound shear wave elastography.
2024	Sheng Xu (37)	Transcranial volumetric imaging using a conformal ultrasound patch.

### 6.2 Artificial intelligence and deep learning

In recent years, advancements in artificial intelligence (AI) have accelerated revolutionary breakthroughs in the application of ultrasound technology, particularly in the fields of automated image recognition and analysis, prediction models, and decision-making support. The application of AI in the automated recognition and analysis of ultrasound images primarily relies on deep learning techniques. Convolutional neural networks are widely employed for feature extraction and classification in ultrasound imaging, including image quality control, automatic classification of echocardiographic views, segment of cardiac structures, and quantification of cardiac function, effectively identifying pathological changes such as segmental wall motion abnormalities, cardiomyopathy, and pericardial effusion (38, 39). Additionally, AI has been applied to hemodynamic measurements such as left ventricular ejection fraction (LVEF) and left ventricular outflow tract velocity time integral (VTI) (40). It is also used to detect potential pathological changes in LUS, assess ICU-acquired weakness in muscle ultrasound, and evaluate gastric contents and antrum function through gastric ultrasound (41–43).

In recent years, researchers have developed multiple deep-learning models to assess and evaluate cardiac function automatically. Gohar E et al. conducted a validation study of three AI-based, real-time, hemodynamic echocardiographic assessment tools. The study showed that the automatic ejection fraction, velocity time integral, and inferior vena cava tools exhibited good agreement with the POCUS expert for high-quality views (44). Shaikh et al. demonstrated that automation-assisted VTI can provide real-time feedback to correct image acquisition for optimal aortic outflow velocity measurement while decreasing variability (45). Other studies showed that the area under curve for AI-assisted echocardiography in identifying abnormal LVEF (<50%) was 0.98, with a sensitivity of 92.8% and a specificity of 92.3% (46). Additionally, Cohen’s Kappa coefficient reached 1.0, indicating perfect agreement (47). The assessment of LVEF by medical students with the help of AI tools was highly consistent with the evaluations made by cardiologists (48).

AI-based LUS can effectively reduce the subjectivity in the interpretation process and serve as an important aid in improving clinical workflow. Tan et al. used a LUS B-line detection AI system to identify fluid overload in dialysis patients. Their study showed a strong correlation and good agreement on B-line count between physicians and AI in both training and validation sets (49). Subsequent studies have also demonstrated that the accuracy, sensitivity, and specificity of AI algorithms for the detection of A-lines and B-lines can all reach over 80% (50, 51). A team from the University of Leeds employed deep learning techniques for real-time multi-class segmentation of lung ultrasound images and developed a strategy for measuring disease severity by automatically calculating the percentage of intercostal space occupied by B-lines (52). Kuroda, Y et al. further investigated the feasibility of AI-assisted LUS for the diagnosis of pulmonary diseases. Their study revealed that AI-assisted LUS in a 12-zone assessment achieved an accuracy of 94.5%, with a sensitivity of 92.3% and a specificity of 100% in detecting COVID-19 pneumonia confirmed by CT. When the assessment was simplified to 8 zones, the accuracy, sensitivity, and specificity were 83.9, 77.5, and 100%, respectively (with a confidence interval of 80.6 to 100%) (53). In addition, Nhat et al. developed a software that can automatically identify and measure the cross-sectional area of the rectus femoris muscles (43). This achievement makes it more feasible to monitor muscle status in ICU patients and holds promise for establishing muscle ultrasound assessment as a routine clinical practice in the ICU.

Despite the remarkable progress in AI-assisted medical image analysis, several key limitations must be acknowledged. First, there are still limited studies on the real-world clinical application of AI in healthcare. The diagnostic performance of AI tools often exhibits variability across different clinical settings and may suffer from performance degradation in real-world applications, as their training datasets may not fully represent diverse patient populations, imaging protocols, or disease manifestations (54). Second, users require clear guidelines and training to manage uncertainties and challenges introduced by AI-driven workflows (55). Third, when AI-assisted decisions lead to adverse outcomes, the allocation of responsibility among clinicians, developers, and healthcare institutions remains legally ambiguous, potentially discouraging adoption. These ethical-legal gaps must be addressed through international consensus frameworks before AI can achieve sustainable integration into high-stakes critical care environments (56). Furthermore, for the full potential of AI-enabled technologies to be successfully implemented and ultimately contribute to the transformation of health systems in the region, foundational investments are needed in digital infrastructure, technology governance, and data governance (57).

It is also important to recognize that current AI-assisted measurements are mainly based on images, necessitating caution when using them to confirm specific diagnoses. Future advancements must integrate multi-dimensional information such as the patient’s abnormal signs, underlying diseases, and laboratory indicators to optimize AI-assisted ultrasound systems. Additionally, the “black-box” nature of many deep learning models raises concerns regarding interpretability, as clinicians may hesitate to trust AI-generated recommendations without transparent decision-making pathways. Addressing this limitation, Yao et al. proposed an AI-generated content-enhanced computer-aided diagnosis model, which enables human-computer interaction and synthesizes multi-source data, such as physician reports, international guidelines, research literature, and ultrasound images. This approach may redefine next-generation AI-assisted diagnostic systems (58). In summary, future research should prioritize multidisciplinary collaborations involving policymakers, ethicists, and clinicians to establish robust validation frameworks and adaptive governance models. Ensuring transparency, safety, and alignment with clinical workflows will be critical to achieving meaningful AI integration in critical care (54) (Table 3).

**Table 3:** Ultrasonographic applications of artificial intelligence.

Year	Researcher	Manufacturer/Model	Application
2021	Maheshwarappa et al. (47)	GE	Automatic calculation of LVEF based on AI system.
2022	Shaikh et al. (45)	GE	Automated VTI measuring system.
2022	Tan et al. (49)	Nanyang polytechnic	Automated lung ultrasound image assessment using AI to identify fluid overload in dialysis patients.
2023	Xing et al. (51)	Faster R-CNN	Automatic detection of A-line in lung ultrasound images using deep learning and image processing.
2023	Gohar et al. (44)	GE	AI-based, real-time, hemodynamic echocardiographic assessment tools.
2023	Motazedian et al.(46)	EchoNous	AI-assisted assessment of LVEF.
2023	Dadon et al. (48)	DiA imaging analysis	AI-based tool with a hand-held ultrasound device for LVEF assessment.
2023	Kuroda et al. (53)	Philips	AI-based point-of-care lung ultrasound for screening COVID-19 pneumonia.
2024	Nekoui et al. (50)	ExoLungAI	AI-based tool for the detection and quantification of A-lines and B-lines.
2024	Howell et al. (52)	U-Net	Deep learning for real-time multi-class segmentation of artifacts in lung ultrasound.
2024	Nhat et al. (43)	RAIMUS	AI-assisted muscle ultrasound for monitoring muscle wasting in ICU patients.

### 6.3 Education and training

As the concept of CCUS continues to evolve, it has become widely used in the healthcare field. However, the implementation and interpretation of CCUS are inherently operator-dependent, and the results of ultrasound examinations are significantly influenced by the operator’s skills and experience in image interpretation. Therefore, it is essential to standardize the use of CCUS to enhance the quality of healthcare practices.

The European Society of Intensive Care Medicine (ESICM) has recommended that CCUS training should focus on the following three areas: (1) general critical care ultrasound, (2) basic critical care echocardiography, and (3) advanced critical care echocardiography (59). Since then, the ESICM has published the core CCUS skills that critical care physicians should master, along with a foundational document outlining CCUS training objectives (60, 61). Despite the clearly defined training standards for CCUS, achieving consistency in the quality of images acquired by operators and their ability to interpret these images continues to pose a significant challenge in CCUS training.

**Figure 4:**
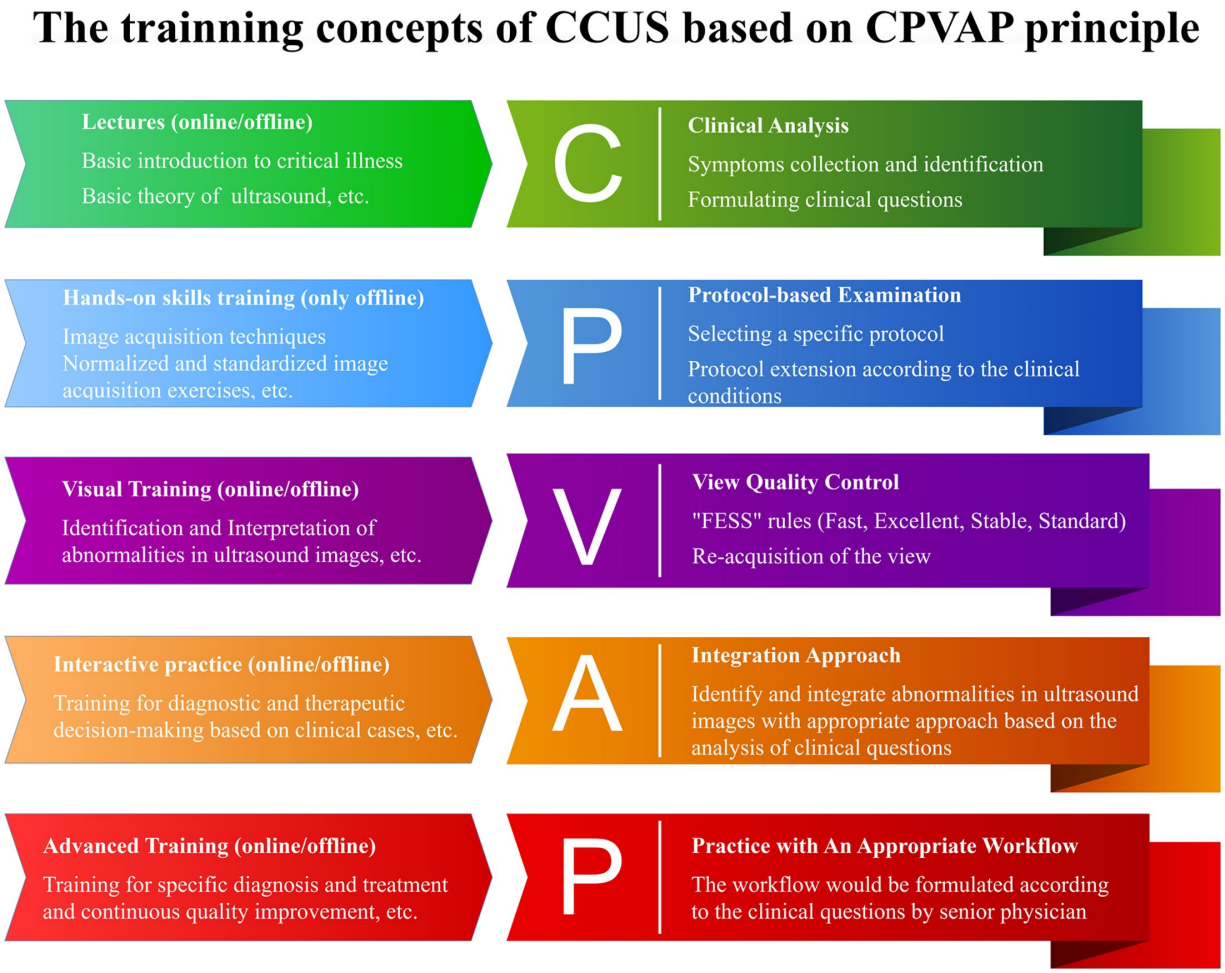
The CPVAP principle.

**Figure 5:**
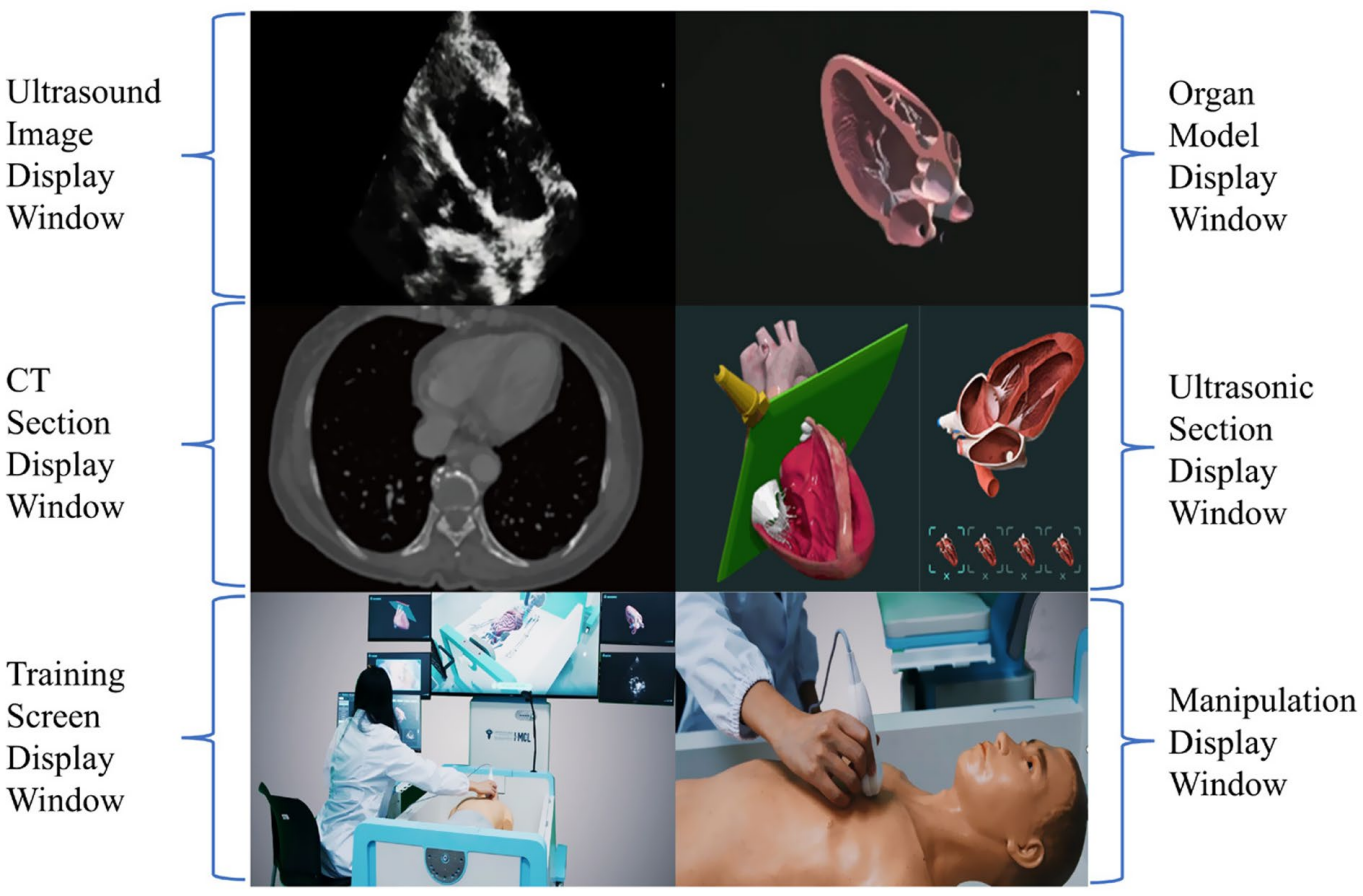
Design of training system.

Yin, et al. thus proposed a systematic training program grounded in the CPVAP principle, which encompasses clinical analysis, protocol-based examination, view quality control, integration approach, and practice workflow (Figure 4) (62). This innovative framework aims to advance the training of CCUS and the visualization of ward rounds. They established a visualization teaching and training center that leverages AI and virtual reality technology and developed a three-dimensional virtual demonstration system equipped with thousands of teaching videos and clinical cases designed by panels of experts. This system consists of three modes: teaching, practice, and assessment. Each mode is further divided into three major modules, which include: (1) image acquisition and quality control skills, (2) visual accumulation training for the interpretation and analysis of ultrasound images, and (3) training focused on diagnosis and treatment decision-making, utilizing standardized clinical cases. This structured approach ensures that learners not only acquire technical skills but also develop critical thinking and decision-making capabilities in real clinical scenarios (62). It can also display ultrasound images, corresponding anatomical images, CT images, ultrasound sections, manipulation techniques, and training scenarios (Figure 5). This multi-faceted approach enriches the learning environment and facilitates a deeper understanding of the ultrasonic views, thus improving overall clinical competence.

## 7 Clinical impacts and outcome evidence of critical care ultrasound

Previous studies indicate that ultrasound application modifies treatment decisions in 39–69% of cases (63–66), with earlier identification of etiology and life-threatening conditions. Patients managed with ultrasound guidance demonstrated reduced fluid overload (65–67), more targeted antibiotic use, and lower radiation exposure (68). However, evidence supporting the impact of CCUS on clinical outcomes remains limited. Existing studies on LUS scores for prognostic prediction show conflicting results, with variations across populations and inconsistent association strengths (69–72). Some evidence suggests that CCUS may reduce mortality by 21% for volume management in acutely ill patients (73), prevent morbidity and mortality in 45% of cases in which it was not used (74), and decrease ventilator days in geriatric resuscitation (75). However, a multicenter randomized trial found that while ultrasound-guided management in critically ill patients with hemodynamic shock or respiratory failure frequently altered diagnoses and therapy, it did not improve 30-day survival (76).

## 8 Critical care ultrasound in perspective: comparative advantages and limitations

While pulmonary artery catheterization remains the gold standard for hemodynamic assessment in cardiogenic shock, it carries inherent risks, including vascular injury, infection, and mechanical complications. In contrast, CCUS offers a noninvasive, multiparametric alternative for comprehensive hemodynamic evaluation, significantly reducing data redundancy with real-time image information (77). In addition to its convenience, reduced costs, and reduced radiation exposure, LUS demonstrates superior diagnostic accuracy for heart failure compared to conventional chest radiography (78–80). In contrast to CT imaging, CCUS provides superior bedside availability and repeatability, with reduced risk related to the transportation of patients to the radiology department and the overall contamination risk. However, CCUS cannot fully replace CT’s comprehensive anatomical evaluation.

## 9 Current limitations of critical care ultrasound

### 9.1 Operator dependence and skill variability

Despite its proven clinical utility, CCUS implementation is constrained by several limitations. Although recent technological advancements have simplified its operation, CCUS still requires extensive training in both image acquisition and interpretation. Standardized protocols are essential to obtain reliable ultrasound images and data, which are critical for objective clinical assessment. However, significant inter-operator variability in measurements, parameter selection, and data interpretation limits the consistency and effectiveness of CCUS in practice.

### 9.2 Equipment and resource limitations

Economic limitations represent a significant barrier to CCUS adoption, particularly in low-resource settings where substantial equipment costs and budget priorities limit accessibility. The rapid iteration of ultrasound technology requires sustained capital investment for equipment renewal and software upgrades.

### 9.3 Controversy over clinical application

CCUS application carries risks of over-reliance, as some clinicians may make decisions solely based on ultrasound findings while neglecting comprehensive clinical assessment. Ultrasound imaging in certain clinical scenarios, particularly in obese patients and post-thoracotomy cases, often yields suboptimal image quality. Consequently, a multimodal diagnostic approach incorporating complementary imaging modalities becomes essential for comprehensive patient assessment.

## 10 Conclusion

The emergence of CCUS marks a significant advancement in ultrasound technology, heralding a new era in CCM. By facilitating the visualization of pathophysiological conditions and enabling standardized ward rounds, CCUS provides a more refined approach to the management of critically ill patients. The introduction of wearable ultrasound devices and the incorporation of AI have expanded CCUS’s potential applications. To ensure the sustainable and effective development of CCUS, the establishment of standardized training programs and uniform quality control measures is essential. Continued research into the clinical applications of CCUS will further clarify its role in CCM, while robust evidence-based guidelines will be vital for optimizing its use across various clinical settings. We remain optimistic that innovations in ultrasound technology, coupled with interdisciplinary collaboration, will boost the prospects of CCUS for better health.
